# Payment for Notification

**Published:** 1898-04-16

**Authors:** 


### PAYMENT FOR NOTIFICATION.

We are sorry to find that Dr. Newsholme, medical officer of health for Brighton, has again been airing his opinion that the fee for notification should be 1s. all round, instead of, as at present, 2s. 6d. for private practitioners, and 1s. for those engaged in public practice. We, on the other hand, would urge that the giving of these certificates is a very onerous and responsible piece of work, involving the exercise of a high degree of special skill; and that, instead of reducing the fee to a shilling, it would be far more equitable to raise it, so as to make it half-a-crown all round. It must be remembered that the medical officer of health is not called upon to visit these cases and ascertain what is the matter with them, and that the whole machinery for the suppression of infectious diseases hinges on the correct diagnosis and early notification of the individual cases by the medical attendant. Unfortunately we see traces on every side of the tendency which pervades the official mind to make the general practitioner trot round for the benefit of the State. This is an entirely wrong position to take up. If the State chooses to institute domiciliary medical visits with the object of ascertaining the presence of infectious disease, and if the people choose to put up with such visitations, well and good; but so long as the authorities ask for and make use of the expert opinions of private practitioners, so long ought they to pay for them, and pay for them well, for the notification certificate is the very pivot on which, so far as infectious diseases are concerned, the sanitary machine revolves.

